# The Fallopian tube: Biosensor and dynamic regulator of preimplantation development

**DOI:** 10.1093/pnasnexus/pgag276

**Published:** 2026-08-14

**Authors:** Kalli K Stephens, Jiude Mao, Wipawee Winuthayanon

**Affiliations:** Division of Animal Sciences, College of Agriculture, Food and Natural Resources, University of Missouri, Columbia, MO 65211, USA; Department of Obstetrics, Gynecology, and Women's Health, School of Medicine, University of Missouri, Columbia, MO 65211, USA; Division of Animal Sciences, College of Agriculture, Food and Natural Resources, University of Missouri, Columbia, MO 65211, USA; Department of Obstetrics, Gynecology, and Women's Health, School of Medicine, University of Missouri, Columbia, MO 65211, USA

**Keywords:** assisted reproductive technologies, embryo-maternal communication, embryo quality, Fallopian tube, oviduct

## Abstract

The Fallopian tube (also called the oviduct) provides the physiological site for fertilization and early embryonic development in mammals, yet its role in shaping preimplantation developmental competence remains incompletely understood. Although assisted reproductive technologies (ARTs) have enabled millions of successful pregnancies, most ART procedures bypass the oviduct, eliminating early embryo-maternal interactions that occur in vivo. Evidence across mammalian species demonstrates that the oviduct is not a passive conduit, but a dynamic tissue that regulates embryo transport, modulates immune and metabolic responses, and secretes stage-specific factors that influence embryonic development. Disruption of oviductal signaling impairs preimplantation development and leads to early pregnancy loss, highlighting the importance of a functional oviductal environment. Moreover, embryos actively send signals to the oviduct, eliciting stage-specific transcriptional and proteomic responses, indicating reciprocal communication during early pregnancy. Data suggest that embryos generated by different ART approaches (such as somatic cell nuclear transfer or artificial insemination) exhibit distinct molecular and metabolic profiles, raising the possibility that embryo source and quality may uniquely shape uterine epithelial responses following transfer. In this review, we provide evidence supporting the oviduct as an active regulator of preimplantation embryonic development, discuss the consequences of bypassing the oviduct in ART, and highlight current experimental models and knowledge gaps. Defining how the oviduct functions as a biosensor of embryo quality will be critical for understanding early pregnancy loss and refinements for ARTs.

## Introduction

In humans, the structure connecting the ovary and the uterus is called the Fallopian tube, or the oviduct in animals. In humans and nonhuman primates, the oviduct is bowed or straight, while in rodents and most other mammals, it is coiled. The degree of coiling varies among species and may indicate functionality and contribution to reproductive success. In rodents, the membranous structure surrounding the ovary and the distal portion of the oviduct, called the bursa, facilitates the capture of ovulated eggs into the oviduct. In contrast, the bursa is absent in humans and non-human primates, allowing peritoneal content to mix with reproductive tract fluid. Despite gross anatomical differences, overall cellular composition, such as secretory and ciliated epithelial, stromal, immune, and endothelial cells, remains the same (1, 2).

The Fallopian tube generally comprises the infundibulum, the ampulla, the isthmus, and the uterotubal junction. In mammals, the embryo resides and develops within the oviduct for 3–6 days, depending on the species (3), before transport to the uterus for implantation. Therefore, a functional oviduct is required for fertilization and the preimplantation embryonic developmental process in vivo. As such, tubal ligation and salpingectomy are effective and permanent contraceptive methods for women. Likewise, pathological conditions of the oviduct, including tubal occlusion or damage from infection, can impede pregnancy. In the United States, tubal factor infertility due to structural or functional oviduct pathology contributes to ∼10.2% of couples seeking fertility treatment (4). While most pregnancy losses happen during implantation, which occurs when the embryo has reached the uterus (5, 6), these losses may be associated with inadequate embryonic development, resulting in implantation failure. This underscores the significance of both embryo quality and oviduct function in establishing a pregnancy. This review examines whether the oviduct functions as an active biosensor that detects embryo quality and adapts its response to support preimplantation development.

## Benefits of the oviduct in assisted reproductive technologies

**Figure 1:**
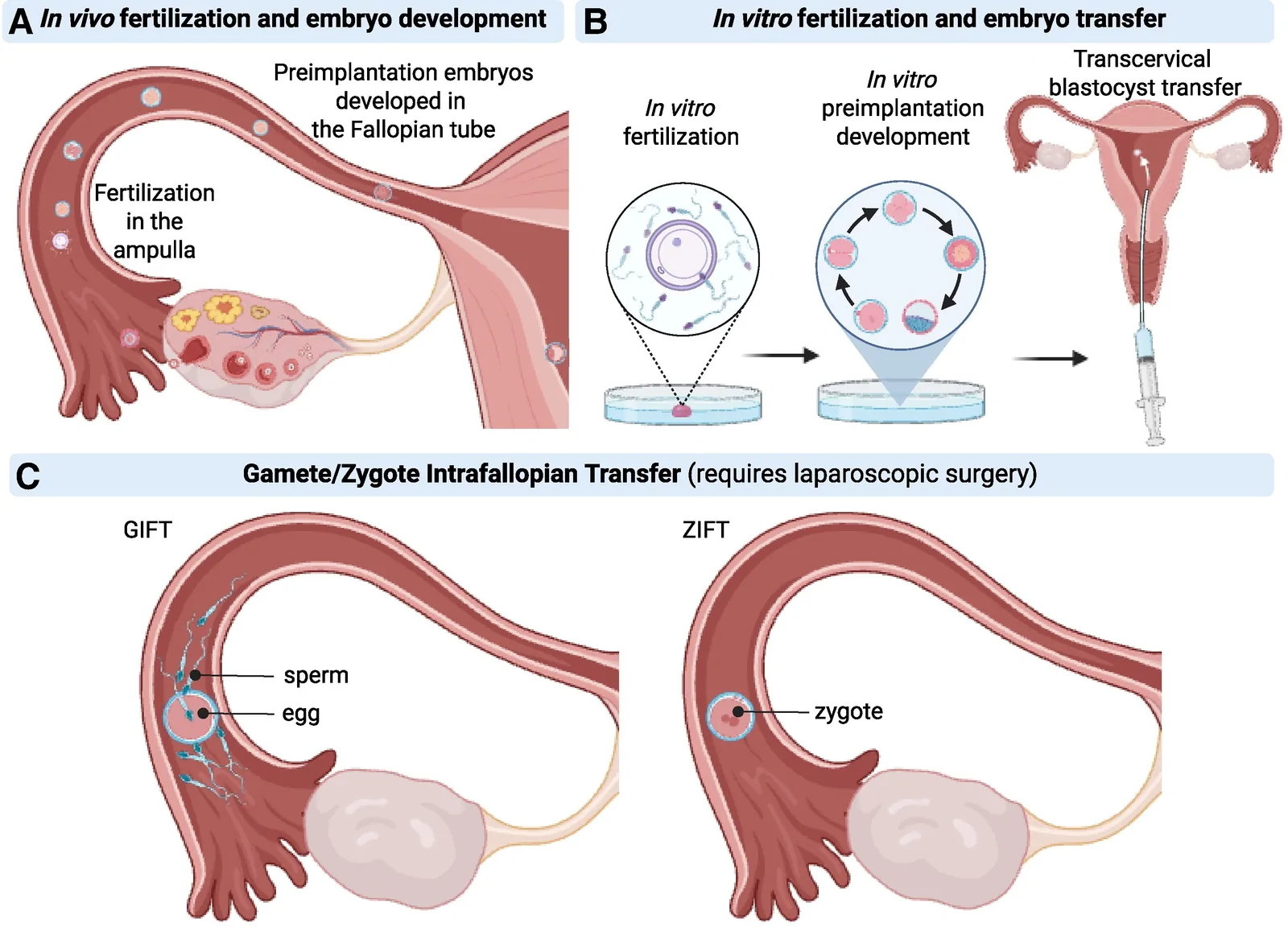
Roles of the oviduct in vivo compared to ARTs. A) A diagram depicting an in vivo fertilization and embryonic development in the Fallopian tubes in humans. Fertilization takes place in the ampulla region, and preimplantation embryos develop in the Fallopian tube. B) In vitro fertilization and preimplantation embryonic development in a culture dish until the blastocyst stage. Then, blastocysts are transcervically transferred into the uterus. C) Gamete/zygote intrafallopian transfer (GIFT/ZIFT) procedures using laparoscopic surgery to directly place gametes (eggs/sperm) or zygotes into the Fallopian tubes. Created in BioRender.

The oviduct connects the ovary to the uterus and is the location of gamete transport, fertilization, and preimplantation development (Fig. 1A). Assisted reproductive technologies (ARTs) are becoming increasingly popular as women choose to have children later in life and couples seek medical assistance to overcome biological fertility barriers associated with advancing maternal age (7, 8). During most ARTs, the Fallopian tube is bypassed. In vitro fertilization-embryo transfer (IVF-ET) is one of the most frequently used techniques for infertile couples. For IVF-ET, eggs and sperm are combined for fertilization in the dish and cultured until the blastocyst stage (Fig. 1B). Then, blastocysts are transferred directly into the uterus. In addition to IVF-ET, gametes or zygotes (fertilized eggs or 1-cell embryos) are transferred into the Fallopian tube instead of the uterus (Fig. 1C). Gamete or zygote intrafallopian transfer (GIFT or ZIFT) allows for in vivo fertilization and/or Fallopian tube exposure during preimplantation embryonic development. However, GIFT is only feasible in patients with functional Fallopian tubes and normal sperm parameters, and is therefore restricted to a subset of couples. Although GIFT/ZIFT were more commonly used in the early era of ART, their use has become obsolete because the procedure requires laparoscopic surgery, whereas conventional IVF does not. While the procedure is offered by some clinics, its use is not high enough to be tracked nationally in the United States.

Early clinical studies comparing GIFT and IVF-ET reported variable outcomes. Higher pregnancy and delivery rates are observed with GIFT compared to IVF protocols. For example, one study reported pregnancy rates of 44.2% with GIFT compared to 31.0% with IVF, with corresponding delivery rates of 33.4 and 22.9%, respectively (9). Mills et al. (10) also found that pregnancy rates in GIFT were 40%, compared with 28% in IVF. Similarly, Dawood reported a pregnancy rate per cycle of 29.5% following GIFT, whereas the average pregnancy rate for IVF in the same clinical setting was 19.8% (11). Similarly, ZIFT also results in higher implantation, clinical pregnancy, and delivery rates compared to IVF-ET in a small nonrandomized study of patients with repeated implantation failure (12). In contrast, a prospective randomized study found no significant differences in pregnancy or live-birth outcomes between GIFT and IVF-ET (13). In addition, GIFT has not been consistently shown to confer a clear advantage over IVF in terms of pregnancy or live-birth outcomes (14). Concurrently, substantial advances in embryo culture conditions, laboratory techniques, and embryo selection have led to marked improvements in IVF success rates, further reducing the clinical use of GIFT/ZIFT.

Overall, these findings suggest that while GIFT/ZIFT was associated with favorable outcomes in some clinical contexts, results varied depending on study design, patient selection, and the technical limitations of early IVF protocols, rather than providing definitive evidence of superiority over IVF-ET. Nevertheless, these observations highlight the oviduct as a critical yet largely bypassed physiological environment during early embryonic development. With current advances in tissue engineering, the roles of the oviduct in human IVF and ET should be revisited and re-evaluated.

## The oviduct is not just a passive conduit for embryos

**Figure 2:**
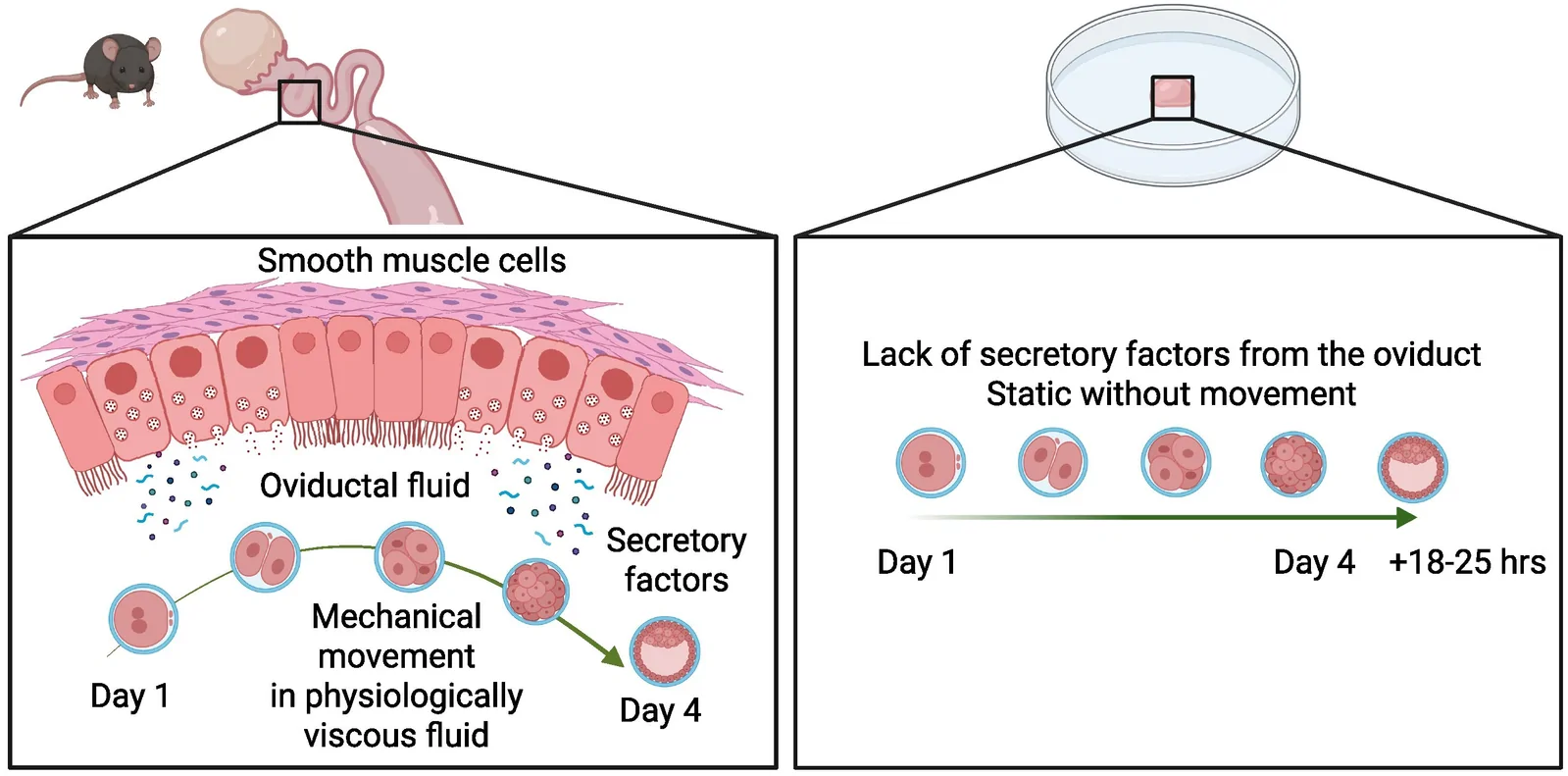
Preimplantation embryonic development in vivo vs. in vitro. In mice, preimplantation embryonic development takes ∼4 days. However, this process requires an additional 18–25 h in vitro for embryos to develop to the blastocyst stage. In the oviduct, the embryos are bathed in the viscous oviductal fluid, which contains factors secreted by the secretory epithelial cells. In addition, the embryos move back and forth, with the net direction toward the uterus, due to the activity of smooth muscle and ciliated epithelial cells. In contrast, embryos are cultured in vitro in a static environment, without physical movement. Created in BioRender.

Even though preimplantation embryos can be developed in vitro, research in various mammalian species has demonstrated that the oviduct is more than just a passage for the embryo before it enters the uterine cavity. Poor conditions within the oviduct may create an unfavorable environment for the preimplantation embryo. Studies in pigs, cows, and rodents show that embryos develop more slowly in vitro culture than in vivo (15–19). For example, in mice, 2-cell embryos take ∼18–25 h longer to develop to the blastocyst stage compared to those developed in vivo (20) (Fig. 2). Moreover, back-and-forth motion of luminal contents and embryos due to peristaltic contraction is observed in vivo in multiple species (21–23). This fluid movement is lacking in the current IVF setting. A more recent study in bovine embryos demonstrates that a gentle flow, created using an in vitro oviduct-on-a-chip microfluidic device, may be crucial for genetic reprogramming of the embryos (24). Ferraz et al. (24) show that zygotes developed in the presence of physical fluid flow in the oviduct-on-a-chip exhibit global demethylation, as measured by 5-methylcytosine, at a level similar to that of in vivo*-*derived zygotes, in contrast to embryos cultured without flow.

In addition to the fluid flow, bovine embryos cultured in media with a viscosity similar to oviductal fluid (∼3.3 mPa·s) have fewer apoptotic cells and lower methylation rates in the inner cell mass than those cultured in lower-viscosity media (25). In bovine models, using a collagen gel coating on the culture plate increases blastocyst rate and expansion compared with culture in standard polystyrene dishes (26). In mice, the use of polydimethylsiloxane (PDMS) compared to standard polystyrene culture dishes increases hatching frequencies and total cell number, while culture with collagen gel improves embryo progression to 2-cell, blastocyst, and hatching stages, and increases trophectoderm cell number (27). This suggests that the substrate used for embryo culture may affect their development. These studies show that a physical movement in the proper viscous oviductal fluid and substrates on the culture dish are crucial for embryonic developmental trajectories, indicating that the physiological condition of the oviduct determines embryonic developmental outcomes.

In mice, a lack of proper estrogen signaling due to the loss of estrogen receptor α (ESR1) expression in oviduct epithelial cells leads to complete embryonic death before the 2-cell stage, resulting in female sterility (28). Winuthayanon et al. (28) show that excess protease activity in female mice with the oviductal epithelial *Esr1* deletion is harmful to developing embryos, and that protease inhibition can rescue embryonic development in vivo. Similarly, the loss of the progesterone receptor in oviductal epithelial cells leads to increased recruitment of macrophages in the oviduct, which could indirectly hinder the development of preimplantation embryos in mice (29). These findings indicate that proper steroid hormone action and signals within the oviduct govern the normal preimplantation embryonic development and prevent early pregnancy loss. Moreover, female mice lacking histone demethylase (*Kdm4a*^−/−^) are severely subfertile due to a preimplantation embryonic developmental defect (30). *Kdm4a* transcripts are expressed throughout the female reproductive tract and in the oocyte (30). To distinguish the contribution of the maternal reproductive tract from that of the oocyte, 2-cell *Kdm4a*^−/−^ embryos were transferred to wild-type recipients. The partial rescue of live *Kdm4a*^−/−^ pups suggests that maternal KDM4A in the reproductive tract contributes to preimplantation embryonic development, although an embryo-intrinsic requirement remains. As KDM4A is a histone demethylase, these findings further suggest that proper epigenetic regulation is required during early embryonic development. This work further demonstrates that, as another factor in the oviduct, maternal expression of histone demethylase is also required for preimplantation embryonic development.

In pigs, cows, and sheep, co-culture of embryos with oviduct epithelial cells improved fertilization rates, blastocyst development, and hatching ability (31–36) (see more details on co-culture below). This beneficial effect on embryos appears to be oviduct-specific, as culture with fibroblasts did not improve embryonic development as much as culture with oviduct epithelial cells (37). Additionally, co-culture with oviductal epithelial cells improved embryo cleavage rate more than co-culture with uterine or renal cells (38). These data indicate that the oviduct provides an optimal microenvironment for the survival/development of the embryo and positively directs its developmental trajectory.

## Reciprocal embryo-oviduct communication during preimplantation development

**Figure 3:**
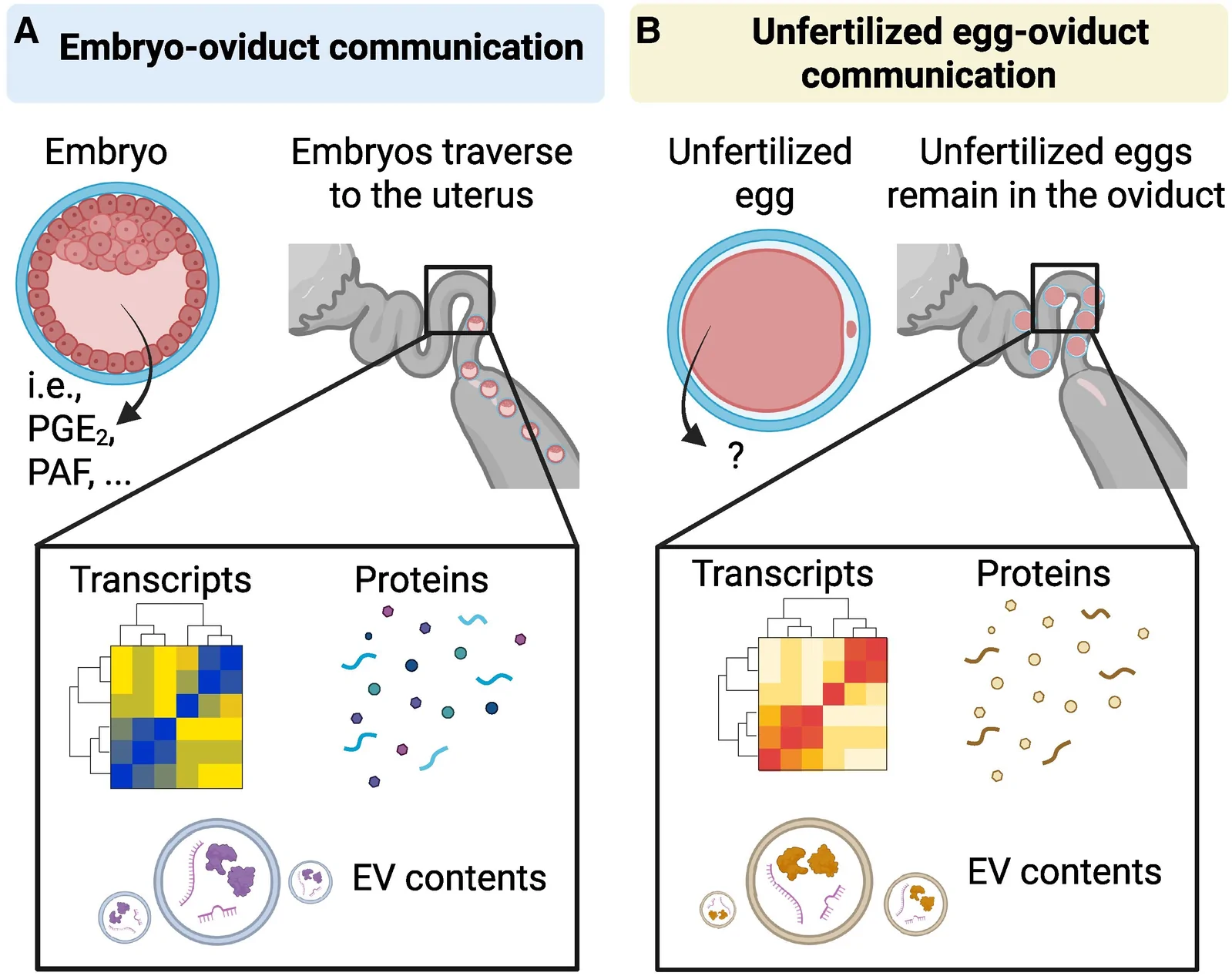
Embryo- vs. unfertilized egg-oviduct communication. A) In multiple species, embryos secrete factors such as prostaglandin E_2_ (PGE_2_) or PAF, which have been shown to be crucial for embryo transport from the oviduct to the uterus. B) In the presence of unfertilized eggs, eggs remain in the oviduct. In addition, the presence of embryos stimulates gene expression profiles, luminal proteins, and EV contents in the oviduct, which are distinct from those of unfertilized eggs. Created in BioRender.

In addition to communication from the maternal oviduct to the embryos, reciprocal dialogue from the embryo and the oviduct exists in several mammalian species (39–43). One example is that fertilized vs. unfertilized eggs are transported at different speeds, which is controlled by oviductal contraction. In horses, blastocysts, but not unfertilized eggs, selectively traverse the oviduct due to embryo-derived prostaglandin E_2_ (39–41). In rats (42) and hamsters (43), unfertilized eggs travel through the oviduct at a different rate compared to embryos (Fig. 3A). In cows, the presence of the embryos significantly decreased oviductal fluid flow (measured by particle transport rates) compared to areas where embryos are not present (44). These findings suggest that distinct signals are produced specifically by the embryos to manipulate the biological function of the oviduct during early pregnancy.

In humans, embryos produce platelet-activating factor (PAF, a potent phospholipid mediator) (45), and its receptor protein is highly expressed in the oviduct epithelial and stromal cells (46). In hamsters, pharmacological inhibition of PAF significantly reduced embryo transport rate in the oviduct (47). These different transport rates appear to be unique and intrinsic states of unfertilized eggs vs. embryos (43). These suggest that intrinsic factors from embryos/eggs control oviductal responsiveness to reciprocally modulate their transport speed and perhaps developmental potential, as mentioned above from the oviduct-on-a-chip study.

In addition to the transport speed, unfertilized eggs vs. embryos induce distinct transcriptional responses in the oviduct. In cows (48, 49), pigs (50), and horses (51), the presence of embryos, but not eggs, altered the expression of genes that are related to the immune response in the oviduct. In mice, one of the genes upregulated specifically in the presence of embryos is thymosin beta 4, which is involved in actin polymerization (52). It was postulated that increased thymosin beta 4 production may facilitate ciliation of oviduct epithelial cells after embryos are transported to the uterus.

Recent studies demonstrate that the mouse oviduct is also a dynamic tissue that responds to the presence of embryos vs. unfertilized eggs (pregnant vs. pseudopregnant) by generating distinct transcriptional and translational profiles unique to pregnancy (53, 54) (Fig. 3A and B). In mice, the luminal protein content in the oviduct during estrus (nonpregnant) differs from that present at early pregnancy stages (53). Finnerty et al. show that genes enriched for pro-inflammatory response are present at 0.5 days post coitus (dpc) and then subside at 1.5 dpc. At 1.5 and 2.5 dpc, genes involved in pyruvate and glycolysis are abundant in the oviduct, likely to provide metabolic support for embryos. Moreover, mouse embryos generated in vitro and transferred to the uterus at the cleavage stage (which would normally still reside in the oviduct) result in increased health complications in adult offspring (55). These findings suggest that the uterine environment may be suboptimal for cleavage-stage embryos and further underscore the importance of the oviductal milieu.

Exogenous hormonal ovarian hyperstimulation (superovulation), commonly used in ART to induce ovulation, alters protein content in the oviduct compared to the oviduct without exogenous stimulation. Stephens et al. (54) illustrate that protein cargos in extracellular vesicles (EVs) collected from mouse oviduct luminal fluid have a high abundance of embryo-protectant proteins, i.e. high-mobility group B1 and serine (or cysteine) peptidase inhibitor, which are only enriched in the presence of 2-cell and 4- to 8-cell stage embryos. These findings indicate that the presence of embryos stimulates distinct gene expression and protein secretion, tailoring the biological function of the oviduct to each stage of preimplantation embryonic development. Further research is needed to understand how changes in the oviduct, particularly in gene transcriptions, regulate and benefit embryos in human IVF clinical settings.

## Embryo source and quality may shape oviductal responses

Studies from uterine biology show that the uterus communicates to and promotes embryo growth and survival (56–59). Moreover, the uterus senses and responds uniquely to different types of embryos (in vivo*-* vs. ART-derived) (60, 61). Evidence from porcine studies indicates that the gene expression profiles of in vitro-produced blastocysts differ from those of in vivo-produced blastocysts (62). Therefore, the signaling molecules secreted by these embryos may diverge, leading to subsequent changes in uterine responses. In agreement with this observation, bovine endometrium exhibits different gene expression profiles in response to IVF-conceived compared to somatic cell nuclear transfer (SCNT)-derived embryos or from artificial insemination compared to IVF/SCNT (60, 61). These findings indicate that uterine tissues respond distinctly to embryos derived from different production procedures.

As of now, it is unclear whether the oviduct can detect and respond to embryos in a manner similar to the uterus. Based on findings from uterine research, we can speculate that the oviduct may detect changes in preimplantation embryonic quality, as preimplantation embryos produced in vivo have distinct gene, protein, and metabolic profiles compared to those produced in vitro. In mice and cows, blastocysts produced by IVF exhibit distinct transcriptional profiles compared with in vivo-produced embryos (16, 63–68). Moreover, mouse blastocysts from IVF or intracytoplasmic sperm injection (ICSI) showed distinct gene expression profiles, specifically increased expression of genes involved in mitochondrial dysfunction and metabolic pathways, compared to those from in vivo (16, 64, 65). In addition, 69% of mitochondrial-related genes are aberrantly expressed in IVF-derived blastocysts compared with in vivo*-*derived blastocysts in mice (69). Moreover, human zygotes incubated in either G5 or human tubal fluid culture media exhibit distinct transcriptional profiles, particularly for genes involved in apoptosis, metabolism, protein processing, and cell-cycle regulation (70). It is possible that embryos produced in vivo and in vitro may secrete different signals in varying amounts, depending on their surroundings. These differing signal profiles could lead to distinct responses in oviductal cells and tissues.

Using nanoliquid chromatography coupled with tandem mass spectrometry (nanoLC-MS/MS) and label-free quantification, Banliat et al. (71) show that 85% of proteins are shared between in vivo and in vitro-produced bovine preimplantation embryos. However, distinct proteins are detected only in IVF-derived embryos at all stages, including the 2-cell, 4- to 8-cell, morula, and blastocyst stages. Embryos derived from in vivo sources produce proteins that are involved in carbohydrate metabolism and various cytoplasmic components. In contrast, the proteins that are abundant in IVF-derived embryos before reaching the morula stage are primarily found in the mitochondrial matrix and are associated with ATP-dependent functions. In bovine preimplantation embryos, the overall metabolic profile is similar between in vitro*-* and in vivo*-*derived embryos (72). However, in vitro-produced embryos have significantly higher glucose metabolism as indicated by a 2-fold increase in aerobic glycolysis. Recent analyses show that proteomic and metabolomic profiles of in vitro*-*derived blastocysts are significantly distinct from those of in vivo (73, 74). Lee et al. (73) demonstrate that several metabolic pathways, particularly those involved in amino acid metabolism, are altered during embryo culture. These studies suggest that the ART-associated method may cause molecular changes in preimplantation embryos. This could alter signaling molecules, which in turn might trigger a distinct response in maternal oviductal tissues. However, experiments are needed to confirm this possibility. Although these findings support the possibility of embryo-quality sensing by the oviduct, direct experimental evidence testing differential oviductal responses to ART- vs. in vivo-derived embryos remains limited.

In addition to embryo quality, it was hypothesized that the oviduct may sense embryonic sex, indicating a potential gender-bias mechanism modulated by the female reproductive tract. Almiñana et al. (75) demonstrated that the bovine oviduct responds distinctly when inseminated with X- vs. Y-spermatozoa, as indicated by changes in gene transcriptional profiles. Specifically, the oviduct inseminated with Y-spermatozoa showed a decrease in immune-related genes (Chemokine [C-C motif] ligand 8 and Interferon regulatory factor 7) compared to those with X-spermatozoa (75). However, it remains unclear whether these X- or Y-spermatozoa-induced changes in oviductal genes have functional consequences that influence pregnancy outcomes in vivo. To our knowledge, no study has directly examined the effect of embryo sex (XX vs. XY) on the oviductal response. Therefore, whether the oviduct is capable of sensing the sex of the early embryo is still an open question.

## Consequences of bypassing the oviduct in assisted reproduction

The late-stage zygote undergoes zygotic genome activation before 2-cell cleavage in mice and approximately the 8-cell stage in humans. Then, de novo methylation takes place from the morula to the blastocyst stage. The initiation of these critical events in embryos subsequently determines blastocyst quality and correlates with implantation outcomes (76). In culture, the initial hurdle in ART was a failure of embryos to develop past the 2-cell stage, called “2-cell block.” This 2-cell block can be overcome by removing glucose and adding glutamine to the culture media (77). Biggers et al. (78) showed that culturing mouse oviduct explants could successfully support the development of morulae and blastocysts at the same rate as in vivo. As such, the presence or absence of the oviduct and its luminal component will likely affect the cascade of implantation events in the uterus, which determine whether a pregnancy establishes or results in early pregnancy loss.

We now know that extensive ART procedures performed prior to the preimplantation period lead to developmental abnormalities and an increased risk of long-term metabolic and cardiovascular disorders in mouse offspring, likely as they coincide with a critical period of epigenetic reprogramming during early development (79, 80). Although most ART-conceived children are healthy, evidence shows a small increase in the incidence of imprinting disorders (4.6% compared to 0.8% in naturally conceived children), such as Beckwith-Wiedemann syndrome, which is associated with epigenetic alterations at the *LIT1* and *H19* imprinted loci (81). Consistent with these findings, studies in mice have shown that ART can induce aberrant DNA methylation and histone modification at imprinted loci, selective loss of imprinting in the placenta, and sex-specific epigenetic alterations in the inner cell mass (82–84).

As the preimplantation period is a critical window for epigenetic reprogramming that establishes cell fate and influences later development, several studies have evaluated transcriptomic and chromatin accessibility profiles in ART-derived blastocysts cultured in vitro. Ruggeri et al. (84) show that the inner cell mass of blastocysts produced using IVF exhibits hypermethylation in differentially methylated regions throughout the genome compared to in vivo*-*derived blastocysts. ART procedures also alter the chromatin architecture of embryos when allowed to develop in vitro (85). In contrast, in vitro*-*derived bovine blastocysts allowed to develop in ewe oviducts have a similar transcript profile compared to that of in vivo*-*developed blastocysts (86). As such, attempts to improve ART techniques by mimicking the oviductal environment have been limited due to the lack of research in understanding how the oviduct (i) provides the optimal microenvironment and (ii) is adaptive or passive during preimplantation embryonic development.

## Implications for physiology-informed ART

Multiple experimental strategies have been developed to mimic the oviductal environment during preimplantation development, including co-culture of embryos with oviductal fluid, EVs, epithelial spheroids, organoids, assembloids, and epithelial cells cultured in air–liquid interface (ALI) (24, 87–92). Although these approaches have yielded important insights, each has limitations that still restrict their ability to recapitulate the dynamic, spatially complex in vivo oviductal milieu.

### Fallopian tube fluid

In studies with bovine and porcine models, adding oviductal fluid from the late follicular to early luteal phases (0.1 to 100% v/v) to culture media has been shown to increase fertilization rates, prevent polyspermy, and improve embryo quality (93). While human reproductive fluid has been used for embryo culture (88), tailoring it to meet the specific needs of each developmental stage is challenging. Most studies also retrieve reproductive fluid from the uterus rather than the Fallopian tube, making it difficult to identify unique luminal contents of the Fallopian tube. In addition to the whole reproductive fluid, EVs from the oviductal fluid, from humans (94), rabbits (95), cows (96), and pigs (97), are also used to supplement culture media and shown to overall improve blastocyst developmental rates. Although limited data from mouse models exist (54), the complete profile of Fallopian tube fluid during fertilization, embryonic genome activation, cleavage stage, compaction, and cavitation remains unclear. Consequently, clinical practice still relies on standard one-step or sequential culture media for embryos (93).

### Monolayer epithelial cells, organoids, spheroids, and assembloids

The co-culture of embryos with somatic monolayer feeder cells, such as granulosa cells, endometrial cells, and buffalo rat cell lines, was historically used in human IVF and is still occasionally employed (98–102). However, this co-culture is often reserved for cases of repeated implantation failure, such as women with >3 unsuccessful attempts (103). As the field advances, efforts are being made to replicate the Fallopian tubes, the natural site for preimplantation development, making the use of somatic monolayer cells or feeder cells from other species less advantageous.

2D epithelial monolayer cultures lack the 3D interactions that are integral to oviductal function (104). Oviduct or Fallopian tube organoids are self-organized epithelial cells in spheres cultured in a matrix such as Matrigel (105). These organoids require appropriate responsiveness to fluctuating ovarian hormone profiles, which remains difficult to achieve in vitro. To circumvent this, co-culture models were developed, in which oviduct epithelial organoids grown in matrix are seeded with stromal cells or in a trans-well system, with stroma growing on the bottom of the plate (106). Although the co-culture of organoids and stroma enhances paracrine signaling, it still does not accurately replicate the in vivo cellular arrangement.

In this context, recent advances in 3D “assembloid” systems that integrate epithelial and stromal compartments into self-organizing structures with an epithelial core surrounded by stromal cells represent a promising approach for more faithfully modeling oviductal physiology. Crawford et al. (104) show that Fallopian tube assembloids are responsive to hormone treatment and that ciliated epithelial cells are functional. Assembloids provide enhanced paracrine signaling and have more similarities to in vivo tissues compared to organoid or monolayer models of epithelial cells. Despite these superior characteristics compared to the 2D model or organoid alone, microinjection is still required to access the secretory fluid or to study the cilia inside the luminal core of the assembloids. Porcine and bovine oviductal spheroids have been developed in suspension culture, allowing for an apical-out structural feature (107). These spheroids have functional cilia and secretory activities that can directly interact with sperm and embryos, and have been shown to improve blastocyst developmental rates (89). However, the spheroid model still lacks paracrine signaling from the stroma. Using apical-out assembloids is more advantageous than the traditional apical-in model, especially for co-culturing embryos with assembloids. This approach provides accessible apical membranes and secretory fluid, further enhancing usability.

In another study, human umbilical vein endothelial cells are added to the Fallopian tube epithelial and mesenchymal stem cells to form assembloids (referred to as organoids in the original study) (108). The presence of endothelial cells may be beneficial. However, without systemic circulation, their use may be limited. Likewise, a recent study in a mouse model shows that proper immune cell function in the oviduct is essential for preimplantation embryo survival (29). Therefore, immune cells may also be needed to provide an optimal microenvironment in the co-culture conditions.

### Epithelial ALI

The last model is an ALI culture (basal surface of epithelium in media and apical surface exposed to air), which has been developed using murine, bovine, and porcine oviductal epithelial cells (90). Chen et al. (90) show that zygotes from corresponding species can be developed in oviductal epithelial ALI conditions to the blastocyst stage without embryo culture media. However, it remains unclear whether the absence of media may have a long-term impact on later development stages. The need for advanced in vitro models, including organoids, assembloids, ALI culture reagents, specialized matrices, and microfluidic fabrication platforms, and potentially tubal fluid retrieval, poses practical challenges that limit their widespread adoption.

Regardless of technological advancements, one should also consider the potential impact of anatomy when performing ART, especially the absence of the ovarian bursa, as mentioned above. Peritoneal and reproductive tract fluids can flow through the Fallopian tubes. As such, inflammatory conditions, such as endometriosis or pelvic inflammatory disease, can adversely affect the physiological properties of the Fallopian tubes. For example, women with hydrosalpinx (an inflamed, fluid-filled Fallopian tube) who have undergone IVF have significantly lower rates of implantation and pregnancy outcomes (8.5 and 19.7%, respectively) compared to 13.7 and 31.2% in women without (109). Moreover, co-culture with Fallopian tube fluid isolated from patients with hydrosalpinx leads to decreased fertilization and blastocyst development rates in mice in vitro (110–112). These findings emphasize that optimal Fallopian tube function is necessary for successful embryonic development and pregnancy.

## Future of embryo culture

The presence of Fallopian tube secretions or assembloids demonstrates positive effects on the fertilization process and the development of preimplantation embryos. However, obtaining Fallopian tubes from the mother and subsequently isolating and expanding various cell types into Fallopian tube assembloids can be quite challenging. Moreover, performing biopsies may damage the structure of the Fallopian tubes, potentially affecting the chances of future natural pregnancies. In addition, there may be restrictions on the use of nonself-seeding cells in embryo culture. The next possibility would be to collect skin fibroblasts from women undergoing fertility treatment and induce them into pluripotent stem cells (iPSCs). Then, iPSCs can be differentiated into various Fallopian tube stem cells (113) and generate patient-derived apical-out Fallopian tube assembloids, containing epithelial, stroma, endothelial, and immune cells. Assuming all cell types are validated to have molecular and cellular characteristics similar to those of cells collected from Fallopian tubes in vivo.

Mature egg and sperm should be co-cultured so that fertilization takes place in the presence of these assembloids. Then, the zygote is further developed into the blastocyst stage before the embryo transfer procedure. During the co-culture, defined culture media can be used to best mimic the Fallopian tube environment. Optimal media viscosity (i.e. 3.3 mPa·s), physical motion (gentle back-and-forth fluid flow), and coating-substrate (i.e. PDMS with collagen) on the culture device may further improve embryonic development in the assembloids/embryo culture. This innovative approach could ultimately lead to improved success rates in ART and perhaps, with minimal negative impact on embryos and their developmental trajectories.

## Conclusion

Cumulative evidence in several mammalian species illustrates that the oviduct is not a passive conduit but an active and adaptive tissue that shapes the preimplantation embryonic microenvironment through dynamic embryo-maternal interactions. Despite substantial advances in in vitro models designed to imitate oviductal physiology, a critical gap remains: the developmental and molecular consequences of reintroducing ART-derived embryos into the oviduct in vivo have not been directly evaluated. Determining whether oviductal exposure can modify or restore aspects of embryonic developmental trajectory would provide key mechanistic insight into how early maternal signals influence developmental competence. Although ART has enabled millions of successful pregnancies and the majority of ART-derived babies are healthy, increasing levels of embryo manipulation have been associated with subtle alterations in developmental trajectories and, in some cases, long-term outcomes (114). If embryos generated by different reproductive approaches produce distinct signaling cues that elicit differential oviductal responses, disruption of this reciprocal communication may alter the sequential maternal signaling events that impact preimplantation development. Establishing whether and how the oviduct functions as a biosensor of embryo quality is therefore central to refining physiology-informed ART strategies while maintaining their established safety and effectiveness in vitro.

## References

[1] Ulrich ND, et al 2022. Cellular heterogeneity of human fallopian tubes in normal and hydrosalpinx disease states identified using scRNA-seq. Dev Cell. 57:914–929.e7. 35320732 10.1016/j.devcel.2022.02.017 PMC9007916

[2] McGlade EA, et al 2021. Cell-type specific analysis of physiological action of estrogen in mouse oviducts. FASEB J. 35:e21563. 33818810 10.1096/fj.202002747R PMC8189321

[3] Croxatto HB . 2002. Physiology of gamete and embryo transport through the Fallopian tube. Reprod Biomed Online. 4:160–169. 12470580 10.1016/s1472-6483(10)61935-9

[4] Center_for_Disease_Control_and_Prevention (2024) National ART summary by National Center for Chronic Disease Prevention and Health Promotion (NCCDPHP). https://www.cdc.gov/art/php/national-summary/index.html

[5] Larsen EC, Christiansen OB, Kolte AM, Macklon N. 2013. New insights into mechanisms behind miscarriage. BMC Med. 11:154. 23803387 10.1186/1741-7015-11-154 PMC3699442

[6] Wilcox AJ, et al 1988. Incidence of early loss of pregnancy. N Engl J Med. 319:189–194. 3393170 10.1056/NEJM198807283190401

[7] Tierney K . 2022. The future of assisted reproductive technology live births in the United States. Popul Res Policy Rev. 41:2289–2309. 35874801 10.1007/s11113-022-09731-5 PMC9289087

[8] Lazzari E, Potančoková M, Sobotka T, Gray E, Chambers GM. 2023. Projecting the contribution of assisted reproductive technology to completed cohort fertility. Popul Res Policy Rev. 42:6. 36789330 10.1007/s11113-023-09765-3 PMC9912242

[9] Sayama M, Araki S, Motoyama M, Tamada T, Sato I. 1996. The clinical efficacy of gamete intrafallopian transfer by minilaparotomy versus in vitro fertilization and embryo transfer. J Obstet Gynaecol Res. 22:409–416. 8987320 10.1111/j.1447-0756.1996.tb01049.x

[10] Mills MS, et al 1992. A prospective controlled study of in-vitro fertilization, gamete intra-Fallopian transfer and intrauterine insemination combined with superovulation. Hum Reprod. 7:490–494. 1522191 10.1093/oxfordjournals.humrep.a137677

[11] Dawood MY . 1996. In vitro fertilization, gamete intrafallopian transfer, and superovulation with intrauterine insemination: efficacy and potential health hazards on babies delivered. Am J Obstet Gynecol. 174:1208–1217. 8623848 10.1016/s0002-9378(96)70663-4

[12] Levran D, et al 2002. Prospective evaluation of blastocyst stage transfer vs. zygote intrafallopian tube transfer in patients with repeated implantation failure. Fertil Steril. 77:971–977. 12009353 10.1016/s0015-0282(02)03080-7

[13] Leeton J, Rogers P, Caro C, Healy D, Yates C. 1987. A controlled study between the use of gamete intrafallopian transfer (GIFT) and in vitro fertilization and embryo transfer in the management of idiopathic and male infertility. Fertil Steril. 48:605–607. 2958366 10.1016/s0015-0282(16)59471-0

[14] Anonymous . Chapter 18: Gamete intrafallopian transfer. 2007. Fertil Steril. 87:S61.

[15] Rizos D, et al 2008. Consequences of in vitro culture conditions on embryo development and quality. Reprod Domest Anim. 43:44–50. 18803756 10.1111/j.1439-0531.2008.01230.x

[16] Giritharan G, et al 2007. Effect of in vitro fertilization on gene expression and development of mouse preimplantation embryos. Reproduction. 134:63–72. 17641089 10.1530/REP-06-0247

[17] McKiernan SH, Bavister BD. 1994. Timing of development is a critical parameter for predicting successful embryogenesis. Hum Reprod. 9:2123–2129. 7868684 10.1093/oxfordjournals.humrep.a138403

[18] Magnani L, Cabot RA. 2008. In vitro and in vivo derived porcine embryos possess similar, but not identical, patterns of Oct4, Nanog, and Sox2 mRNA expression during cleavage development. Mol Reprod Dev. 75:1726–1735. 18425776 10.1002/mrd.20915

[19] Corcoran D, et al 2006. Suppressed expression of genes involved in transcription and translation in in vitro compared with in vivo cultured bovine embryos. Reproduction. 131:651–660. 16595716 10.1530/rep.1.01015

[20] Harlow GM, Quinn P. 1982. Development of preimplantation mouse embryos in vivo and in vitro. Aust J Biol Sci. 35:187–193. 7126059 10.1071/bi9820187

[21] Pawliński B, Domino M, Aniołek O, Ziecik A, Gajewski Z. 2016. Bioelectrical activity of porcine oviduct and uterus during spontaneous and induced estrus associated with cyclic hormone changes. Theriogenology. 86:2312–2322. 27590095 10.1016/j.theriogenology.2016.07.029

[22] Han H, Fang T, Mukhamedjanova A, Wang S. 2025. In vivo dynamic imaging reveals the oviduct as a leaky peristaltic pump in transporting a preimplantation embryo toward pregnancy. Biomed Opt Express. 16:3156–3171. 40809967 10.1364/BOE.565065 PMC12339303

[23] Wånggren K, Stavreus-Evers A, Olsson C, Andersson E, Gemzell-Danielsson K. 2008. Regulation of muscular contractions in the human Fallopian tube through prostaglandins and progestagens. Hum Reprod. 23:2359–2368. 18621753 10.1093/humrep/den260

[24] Ferraz MAMM, et al 2018. An oviduct-on-a-chip provides an enhanced in vitro environment for zygote genome reprogramming. Nat Commun. 9:4934. 30467383 10.1038/s41467-018-07119-8 PMC6250703

[25] Fontes PK, Milazzotto MP, Ferraz MdAMM. 2025. Mimicking the oviductal fluid viscosity improves the quality of in vitro-produced bovine embryos. Biol Reprod. 113:1364–1374. 40891494 10.1093/biolre/ioaf202

[26] Rivera RM, Rinaudo P. 2013. Bovine preimplantation embryo development is affected by the stiffness of the culture substrate. Mol Reprod Dev. 80:184–184. 23325633 10.1002/mrd.22152 PMC4121333

[27] Kolahi KS, et al 2012. Effect of substrate stiffness on early mouse embryo development. PLoS One. 7:e41717. 22860009 10.1371/journal.pone.0041717 PMC3409240

[28] Winuthayanon W, et al 2015. Oviductal estrogen receptor alpha signaling prevents protease-mediated embryo death. Elife. 4:e10453. 26623518 10.7554/eLife.10453 PMC4718728

[29] McGlade EA, et al 2025. Progesterone signaling in oviductal epithelial cells modulates the immune response to support preimplantation embryonic development. Sci Adv. 11:eadt6113. 40249812 10.1126/sciadv.adt6113 PMC12007591

[30] Sankar A, et al 2017. Maternal expression of the histone demethylase Kdm4a is crucial for pre-implantation development. Development. 144:3264–3277. 28827393 10.1242/dev.155473

[31] Bavister BD . 1988. Role of oviductal secretions in embryonic growth in vivo and in vitro. Theriogenology. 29:143–154.

[32] Bongso A, et al 1989. Improved quality of human embryos when co-cultured with human ampullary cells. Hum Reprod. 4:706–713. 2778057 10.1093/oxfordjournals.humrep.a136971

[33] White KL, et al 1989. Early embryonic development in vitro by coculture with oviductal epithelial cells in pigs. Biol Reprod. 41:425–430. 2590713 10.1095/biolreprod41.3.425

[34] Zhu J, et al 2016. Human fallopian tube epithelium co-culture with murine ovarian follicles reveals crosstalk in the reproductive cycle. Mol Hum Reprod. 22:756–767. 27542947 10.1093/molehr/gaw041 PMC5099996

[35] Thibodeaux JK, et al 1992. Coculture of in vitro fertilized bovine embryos with oviductal epithelial cells originating from different stages of the estrous cycle. J Dairy Sci. 75:1448–1455. 1500550 10.3168/jds.S0022-0302(92)77900-4

[36] Lorenzo MS, et al 2024. The coculture of in vitro produced porcine embryos and oviductal epithelial cells improves blastocyst formation and modify embryo quality. Theriogenology. 226:141–150. 38885555 10.1016/j.theriogenology.2024.06.007

[37] Gandolfi F, Moor RM. 1987. Stimulation of early embryonic development in the sheep by co-culture with oviduct epithelial cells. Reproduction. 81:23–28. 10.1530/jrf.0.0810023 3668954

[38] Rexroad CE Jr, Powell AM. 1988. Co-culture of ovine ova with oviductal cells in medium 199. J Anim Sci. 66:947–953. 3378953 10.2527/jas1988.664947x

[39] Weber JA, Freeman DA, Vanderwall DK, Woods GL. 1991. Prostaglandin E2 hastens oviductal transport of equine embryos. Biol Reprod. 45:544–546. 1751628 10.1095/biolreprod45.4.544

[40] Robinson SJ, Neal H, Allen WR. 2000. Modulation of oviductal transport in mares by local application of prostaglandin E2. J Reprod Fertil Suppl. (56):587–592. 20681173

[41] Weber JA, Freeman DA, Vanderwall DK, Woods GL. 1991. Prostaglandin E2 secretion by oviductal transport-stage equine embryos. Biol Reprod. 45:540–543. 1751627 10.1095/biolreprod45.4.540

[42] Ortiz ME, Llados C, Croxatto HB. 1989. Embryos of different ages transferred to the rat oviduct enter the uterus at different times. Biol Reprod. 41:381–384. 2590709 10.1095/biolreprod41.3.381

[43] Ortiz ME, Bedregal P, Carvajal MI, Croxatto HB. 1986. Fertilized and unfertilized ova are transported at different rates by the hamster oviduct. Biol Reprod. 34:777–781. 3708057 10.1095/biolreprod34.4.777

[44] Kolle S, et al 2009. Ciliary transport, gamete interaction, and effects of the early embryo in the oviduct: ex vivo analyses using a new digital videomicroscopic system in the cow. Biol Reprod. 81:267–274. 19299315 10.1095/biolreprod.108.073874

[45] Smal MA, Dziadek M, Cooney SJ, Attard M, Baldo BA. 1990. Examination for platelet-activating factor production by preimplantation mouse embryos using a specific radioimmunoassay. J Reprod Fertil. 90:419–425. 2250241 10.1530/jrf.0.0900419

[46] Velasquez LA, et al 2001. PAF receptor and PAF acetylhydrolase expression in the endosalpinx of the human Fallopian tube: possible role of embryo-derived PAF in the control of embryo transport to the uterus. Hum Reprod. 16:1583–1587. 11473946 10.1093/humrep/16.8.1583

[47] Velasquez LA, Aguilera JG, Croxatto HB. 1995. Possible role of platelet-activating factor in embryonic signaling during oviductal transport in the hamster. Biol Reprod. 52:1302–1306. 7632839 10.1095/biolreprod52.6.1302

[48] Schmaltz-Panneau B, et al 2014. Early bovine embryos regulate oviduct epithelial cell gene expression during in vitro co-culture. Anim Reprod Sci. 149:103–116. 25113901 10.1016/j.anireprosci.2014.06.022

[49] Maillo V, et al 2015. Oviduct-embryo interactions in cattle: two-way traffic or a one-way street? Biol Reprod. 92:144. 25926440 10.1095/biolreprod.115.127969 PMC6366479

[50] Almiñana C, et al 2012. Early developing pig embryos mediate their own environment in the maternal tract. PLoS One. 7:e33625. 22470458 10.1371/journal.pone.0033625 PMC3314662

[51] Smits K, et al 2016. The equine embryo influences immune-related gene expression in the oviduct. Biol Reprod. 94:36. 26740593 10.1095/biolreprod.115.136432

[52] Lee KF, Yao YQ, Kwok KL, Xu JS, Yeung WS. 2002. Early developing embryos affect the gene expression patterns in the mouse oviduct. Biochem Biophys Res Commun. 292:564–570. 11906198 10.1006/bbrc.2002.6676

[53] Finnerty RM, et al 2025. Multi-omics analyses and machine learning prediction of oviductal responses in the presence of gametes and embryos. eLife. 13:RP100705. 40009070 10.7554/eLife.100705 PMC11864756

[54] Stephens KK, et al 2024. Proteomic analysis and in vivo visualization of extracellular vesicles from mouse oviducts during pre-implantation embryo development. FASEB J. 38:e70035. 39239798 10.1096/fj.202400041RR PMC11384279

[55] Simbulan RK, et al 2025. Impact of embryo culture and day of embryo transfer on the cardiometabolic health of adult mice. Biol Reprod. 113:657–671. 40448584 10.1093/biolre/ioaf120 PMC12448587

[56] Ng YH, et al 2013. Endometrial exosomes/microvesicles in the uterine microenvironment: a new paradigm for embryo-endometrial cross talk at implantation. PLoS One. 8:e58502. 23516492 10.1371/journal.pone.0058502 PMC3596344

[57] Burns G, et al 2014. Extracellular vesicles in luminal fluid of the ovine uterus. PLoS One. 9:e90913. 24614226 10.1371/journal.pone.0090913 PMC3948691

[58] Bazer FW, Spencer TE, Ott TL. 1997. Interferon tau: a novel pregnancy recognition signal. Am J Reprod Immunol. 37:412–420. 9228295 10.1111/j.1600-0897.1997.tb00253.x

[59] Sandra O, et al 2015. Maternal organism and embryo biosensoring: insights from ruminants. J Reprod Immunol. 108:105–113. 25617112 10.1016/j.jri.2014.12.005

[60] Mansouri-Attia N, et al 2009. Endometrium as an early sensor of in vitro embryo manipulation technologies. Proc Natl Acad Sci U S A. 106:5687–5692. 19297625 10.1073/pnas.0812722106 PMC2667091

[61] Bauersachs S, et al 2009. The endometrium responds differently to cloned versus fertilized embryos. Proc Natl Acad Sci U S A. 106:5681–5686. 19307558 10.1073/pnas.0811841106 PMC2666995

[62] Canovas S, et al 2017. DNA methylation and gene expression changes derived from assisted reproductive technologies can be decreased by reproductive fluids. Elife. 6:e23670. 28134613 10.7554/eLife.23670 PMC5340525

[63] Wang S, Cowan CA, Chipperfield H, Powers RD. 2005. Gene expression in the preimplantation embryo: in-vitro developmental changes. Reprod Biomed Online. 10:607–616. 15949218 10.1016/s1472-6483(10)61668-9

[64] Giritharan G, et al 2010. Effect of ICSI on gene expression and development of mouse preimplantation embryos. Hum Reprod. 25:3012–3024. 20889529 10.1093/humrep/deq266 PMC2989873

[65] Giritharan G, et al 2012. In vitro culture of mouse embryos reduces differential gene expression between inner cell mass and trophectoderm. Reproductive Sciences. 19:243–252. 22383776 10.1177/1933719111428522 PMC3343151

[66] Bridges PJ, et al 2011. Methodology matters: IVF versus ICSI and embryonic gene expression. Reprod Biomed Online. 23:234–244. 21665548 10.1016/j.rbmo.2011.04.007 PMC3151342

[67] Arias ME, Risopatron J, Sanchez R, Felmer R. 2015. Intracytoplasmic sperm injection affects embryo developmental potential and gene expression in cattle. Reprod Biol. 15:34–41. 25726375 10.1016/j.repbio.2014.11.001

[68] Rinaudo P, Schultz RM. 2004. Effects of embryo culture on global pattern of gene expression in preimplantation mouse embryos. Reproduction. 128:301–311. 15333781 10.1530/rep.1.00297

[69] Ren L, et al 2015. Dynamic comparisons of high-resolution expression profiles highlighting mitochondria-related genes between in vivo and in vitro fertilized early mouse embryos. Hum Reprod. 30:2892–2911. 26385791 10.1093/humrep/dev228

[70] Kleijkers SHM, et al 2015. Differences in gene expression profiles between human preimplantation embryos cultured in two different IVF culture media. Hum Reprod. 30:2303–2311. 26202924 10.1093/humrep/dev179

[71] Banliat C, et al 2022. The proteomic analysis of bovine embryos developed in vivo or in vitro reveals the contribution of the maternal environment to early embryo. BMC Genomics. 23:839. 36536309 10.1186/s12864-022-09076-5 PMC9764490

[72] Khurana NK, Niemann H. 2000. Energy metabolism in preimplantation bovine embryos derived in vitro or in vivo. Biol Reprod. 62:847–856. 10727252 10.1095/biolreprod62.4.847

[73] Lee SH, Lira-Albarrán S, Rinaudo PF. 2025. Proteomic and metabolomic insights into oxidative stress response activation in mouse embryos generated by *in vitro* fertilization. Hum Reprod Open. 2025:hoaf022. 40416391 10.1093/hropen/hoaf022 PMC12101870

[74] Zhang H-L, et al 2021. Increased environment-related metabolism and genetic expression in the in vitro matured mouse oocytes by transcriptome analysis. Front Cell Dev Biol. 9:642010. 33681227 10.3389/fcell.2021.642010 PMC7928285

[75] Almiñana C, et al 2014. The battle of the sexes starts in the oviduct: modulation of oviductal transcriptome by X and Y-bearing spermatozoa. BMC Genomics. 15:293. 24886317 10.1186/1471-2164-15-293 PMC4035082

[76] Parks JC, McCallie BR, Janesch AM, Schoolcraft WB, Katz-Jaffe MG. 2011. Blastocyst gene expression correlates with implantation potential. Fertil Steril. 95:1367–1372. 20864103 10.1016/j.fertnstert.2010.08.009

[77] Chatot CL, Ziomek CA, Bavister BD, Lewis JL, Torres I. 1989. An improved culture medium supports development of random-bred 1-cell mouse embryos in vitro. J Reprod Fertil. 86:679–688. 2760894 10.1530/jrf.0.0860679

[78] Biggers JD, Gwatkin RBL, Brinster RL. 1962. Development of mouse embryos in organ cultures of Fallopian tubes on a chemically defined medium. Nature. 194:747–749. 13869115 10.1038/194747a0

[79] de Waal E, et al 2015. The cumulative effect of assisted reproduction procedures on placental development and epigenetic perturbations in a mouse model. Hum Mol Genet. 24:6975–6985. 26401051 10.1093/hmg/ddv400 PMC4654053

[80] Vrooman LA, Bartolomei MS. 2017. Can assisted reproductive technologies cause adult-onset disease? Evidence from human and mouse. Reprod Toxicol. 68:72–84. 27474254 10.1016/j.reprotox.2016.07.015 PMC5269604

[81] DeBaun MR, Niemitz EL, Feinberg AP. 2003. Association of in vitro fertilization with Beckwith-Wiedemann syndrome and epigenetic alterations of LIT1 and H19. Am J Hum Genet. 72:156–160. 12439823 10.1086/346031 PMC378620

[82] Li T, et al 2005. IVF results in de novo DNA methylation and histone methylation at an Igf2-H19 imprinting epigenetic switch. Mol Hum Reprod. 11:631–640. 16219628 10.1093/molehr/gah230

[83] Mann MR, et al 2004. Selective loss of imprinting in the placenta following preimplantation development in culture. Development. 131:3727–3735. 15240554 10.1242/dev.01241

[84] Ruggeri E, et al 2020. Sex-specific epigenetic profile of inner cell mass of mice conceived in vivo or by IVF. Mol Hum Reprod. 26:866–878. 33010164 10.1093/molehr/gaaa064 PMC7821709

[85] Wu J, et al 2016. The landscape of accessible chromatin in mammalian preimplantation embryos. Nature. 534:652–657. 27309802 10.1038/nature18606

[86] Rizos D, et al 2002. Analysis of differential messenger RNA expression between bovine blastocysts produced in different culture systems: implications for blastocyst quality. Biol Reprod. 66:589–595. 11870062 10.1095/biolreprod66.3.589

[87] Menjivar NG, et al 2026. Organoids simulating the bovine oviduct mediate the embryo–maternal interface via extracellular vesicle-transmitted signaling. Hum Reprod Open. 2026:hoaf076. 41503164 10.1093/hropen/hoaf076 PMC12774516

[88] Canha-Gouveia A, et al 2021. Physicochemical and functional characterization of female reproductive fluids: a report of the first two infants born following addition of their mother’s fluids to the embryo culture media. Front Physiol. 12:710887. 34552502 10.3389/fphys.2021.710887 PMC8451538

[89] Pranomphon T, et al 2024. Oviduct epithelial spheroids during in vitro culture of bovine embryos mitigate oxidative stress, improve blastocyst quality and change the embryonic transcriptome. Biol Res. 57:73. 39438935 10.1186/s40659-024-00555-5 PMC11494963

[90] Chen S, et al 2017. An air-liquid interphase approach for modeling the early embryo-maternal contact zone. Sci Rep. 7:42298. 28181558 10.1038/srep42298 PMC5299422

[91] Holthaus D, et al 2025. Establishing and maintaining 3D organoid cultures from human Fallopian tube epithelium. Bio Protoc. 15:e5412. 10.21769/BioProtoc.5412 PMC12378425 40873477

[92] Gardner DK, Lane M. 1998. Culture of viable human blastocysts in defined sequential serum-free media. Hum Reprod. 13:148–160. 9755421 10.1093/humrep/13.suppl_3.148

[93] Hernández-Díaz N, Navarro-Serna S, Coy P, Pérez-García V. 2026. Reproductive fluids, commercial media, and organoids: bridging the gap in IVF culture systems. Hum Reprod Open. 2026:hoag028. 42052577 10.1093/hropen/hoag028 PMC13117623

[94] Li Y, et al 2023. Extracellular vesicles from human Fallopian tubal fluid benefit embryo development *in vitro*. Hum Reprod Open. 2023:hoad006. 36895886 10.1093/hropen/hoad006 PMC9991590

[95] Qu P, et al 2020. Extracellular vesicles and melatonin benefit embryonic develop by regulating reactive oxygen species and 5-methylcytosine. J Pineal Res. 68:e12635. 32012354 10.1111/jpi.12635 PMC7154726

[96] Lopera-Vasquez R, et al 2017. Effect of bovine oviductal extracellular vesicles on embryo development and quality *in vitro*. Reproduction. 153:461–470. 28104825 10.1530/REP-16-0384

[97] de Alcântara-Neto AS, et al 2022. Oviductal extracellular vesicles enhance porcine in vitro embryo development by modulating the embryonic transcriptome. Biomolecules. 12:1300. 36139139 10.3390/biom12091300 PMC9496104

[98] Plachot M, et al 1993. Fertilization and early embryology: granulosa cells improve human embryo development in vitro. Hum Reprod. 8:2133–2140. 8150916 10.1093/oxfordjournals.humrep.a137995

[99] Simón C, et al 1999. Coculture of human embryos with autologous human endometrial epithelial cells in patients with implantation failure. J Clin Endocrinol Metab. 84:2638–2646. 10443653 10.1210/jcem.84.8.5873

[100] Mercader A, et al 2003. Clinical experience and perinatal outcome of blastocyst transfer after coculture of human embryos with human endometrial epithelial cells: a 5-year follow-up study. Fertil Steril. 80:1162–1168. 14607568 10.1016/s0015-0282(03)01178-6

[101] Hu Y, Maxson WS, Hoffman DI, Eager S, Dupre J. 1997. Coculture of human embryos with buffalo rat liver cells for women with decreased prognosis in in vitro fertilization. Am J Obstet Gynecol. 177:358–363. 9290451 10.1016/s0002-9378(97)70198-4

[102] Hu Y, et al 1998. Co-culture with assisted hatching of human embryos using buffalo rat liver cells. Hum Reprod. 13:165–168. 10.1093/humrep/13.1.165 9512251

[103] Benkhalifa M, et al 2012. Autologous embryo–cumulus cells co-culture and blastocyst transfer in repeated implantation failures: a collaborative prospective randomized study. Zygote. 20:173–180. 21473794 10.1017/S0967199411000062

[104] Crawford AJ, et al 2024. Combined assembloid modeling and 3D whole-organ mapping captures the microanatomy and function of the human fallopian tube. Sci Adv. 10:eadp6285. 39331707 10.1126/sciadv.adp6285 PMC11430475

[105] Kessler M, et al 2015. The notch and wnt pathways regulate stemness and differentiation in human fallopian tube organoids. Nat Commun. 6:8989. 26643275 10.1038/ncomms9989 PMC4686873

[106] Qin G, et al 2023. Distinct niche structures and intrinsic programs of fallopian tube and ovarian surface epithelial cells. iScience. 26:105861. 36624845 10.1016/j.isci.2022.105861 PMC9823228

[107] Mahé C, et al 2025. Bovine ampullary and isthmic epithelial spheroids: proteomic profile and physiological features for in vitro studies of gamete-oviduct interactions. Reprod Biol. 25:101057. 40818331 10.1016/j.repbio.2025.101057

[108] Chang Y-H, Chu T-Y, Ding D-C. 2020. Human fallopian tube epithelial cells exhibit stemness features, self-renewal capacity, and wnt-related organoid formation. J Biomed Sci. 27:32. 32035490 10.1186/s12929-019-0602-1 PMC7007656

[109] Camus E, et al 1999. Pregnancy rates after in-vitro fertilization in cases of tubal infertility with and without hydrosalpinx: a meta-analysis of published comparative studies. Hum Reprod. 14:1243–1249. 10325271 10.1093/humrep/14.5.1243

[110] Rawe VJ, et al 1997. Effect of human hydrosalpinx fluid on murine embryo development and implantation. Fertil Steril. 68:668–670. 9341608 10.1016/s0015-0282(97)00319-1

[111] Carrasco I, Cebral E, Benitez R, Vantman D. 2001. Hydrosalpinx fluid affects murine embryonic development in a coculture system with epithelial endometrial cells. Fertil Steril. 75:1004–1008. 11334916 10.1016/s0015-0282(01)01683-1

[112] Thongyou Y, Somsak P, Piromlertamorn W, Sanmee U. 2023. Effect of human hydrosalpinx fluid in fertilization rate of mouse oocyte and embryo quality. JBRA Assist Reprod. 27:20–24. 35257559 10.5935/1518-0557.20210118 PMC10065765

[113] Yucer N, et al 2017. Directed differentiation of human induced pluripotent stem cells into Fallopian tube epithelium. Sci Rep. 7:10741. 28878359 10.1038/s41598-017-05519-2 PMC5587694

[114] Rinaudo P, Simbulan R. 2026. When less really is more: reducing embryo manipulation to lower metabolic stress and improving long term outcomes. Reprod Fertil Dev. 38:RD25163 41291996 10.1071/RD25163 PMC13011073

